# Effective Two-Stage Heterotrophic Cultivation of the Unicellular Green Microalga *Chromochloris zofingiensis* Enabled Ultrahigh Biomass and Astaxanthin Production

**DOI:** 10.3389/fbioe.2022.834230

**Published:** 2022-02-24

**Authors:** Qiaohong Chen, Yi Chen, Quan Xu, Hu Jin, Qiang Hu, Danxiang Han

**Affiliations:** ^1^ Center for Microalgal Biotechnology and Biofuels, Institute of Hydrobiology, Chinese Academy of Sciences, Wuhan, China; ^2^ College of Life Sciences, University of Chinese Academy of Sciences, Beijing, China; ^3^ Institute for Advanced Study, Shenzhen University, Shenzhen, China; ^4^ Key Laboratory for Algal Biology, Institute of Hydrobiology, Chinese Academy of Sciences, Wuhan, China

**Keywords:** Chromochloris zofingiensis, heterotrophic cultivation, biomass, astaxanthin, total fatty acids

## Abstract

*Chromochloris zofingiensis* has obtained particular interest as a promising candidate for natural astaxanthin production. In this study, we established a two-stage heterotrophic cultivation process, by using which both the growth of *C*. *zofingiensis* and astaxanthin accumulation are substantially enhanced. Specifically, the ultrahigh biomass concentration of 221.3 g L^−1^ was achieved under the optimum culture conditions in 7.5 L fermenter during 12 days. When scaled-up in the 500 L fermentor, the biomass yield reached 182.3 g L^−1^ in 9 days, while the astaxanthin content was 0.068% of DW. To further promote astaxanthin accumulation, gibberellic Acid-3 (GA3) was screened from a variety of phytohormones and was combined with increased C/N ratio and NaCl concentration for induction. When *C. zofingiensis* was grown with the two-stage cultivation strategy, the astaxanthin yield reached 0.318 g L^−1^, of which the biomass yield was 235.4 g L^−1^ and astaxanthin content was 0.144% of DW. The content of the total fatty acids increased from 23 to 42% of DW simultaneously. Such an astaxanthin yield was 5.4-fold higher than the reported highest record and surpassed the level of *Haematococcus pluvialis*. This study demonstrated that heterotrophic cultivation of *C. zofingiensis* is competitive for industrial astaxanthin production.

## Introduction

Astaxanthin (3, 3′-dihydroxy-β, β-carotene-4, 4′-dione) is a high-value red ketocarotenoid and possesses a wide range of commercial applications in food, feed, cosmetics, pharmaceuticals, and nutraceuticals due to its powerful antioxidative activity, pigmentation function, and many other bioactivities (Ambati et al., 2014; Liu et al., 2014). Astaxanthin can be synthesized artificially or produced by organisms such as green microalgae. The (3S, 3′S) isoform is the most abundant stereoisomer of the natural astaxanthin produced by green microalgae, whereas the artificially synthesized astaxanthin contains a mixture of isomers (3S, 3′S), (3R, 3S), and (3R, 3′R) at the ratio of 1:2:1 (Li et al., 2020). In addition, the natural astaxanthin produced by microalgae are acylated with one or two fatty acids, whereas the synthesized astaxanthin is free type (Katsumata et al., 2014). Notably, the antioxidant activity of artificially synthesized astaxanthin is 20 times lower than its natural counterpart and is not allowed to apply in human foods in many countries (Rodríguez-Sáiz et al., 2010; Shah et al., 2016). Thus, demands for the natural astaxanthin are increasing and its price reached 7000 US dollars per kg in the global market (Koller et al., 2014). Currently, *H. pluvialis* is the major resource of natural astaxanthin production strain in the industry, as they can accumulate up to 5–6% of astaxanthin on dry weight (Yang et al., 2016). However, astaxanthin production by using *H. pluvialis* is faced with technical bottlenecks such as high cost, low production efficiency, and inevitable contamination by the pathogenic fungus in outdoor mass cultivation (Liu et al., 2013).

One promising way to overcome the techno-economic challenges in natural astaxanthin production is to invent innovative biomanufacturing systems based on discovery and development of novel microalgal species/strains with high growth rates and superior astaxanthin-producing abilities. The green microalga *Chromochloris zofingiensis* is emerging as a promising bioresource for astaxanthin and lipids production (Liu et al., 2014). *C. zofingiensis* can not only utilize light energy and CO_2_ for photoautotrophic growth but also use organic carbon sources so can be cultivated under heterotrophic conditions. The dry cell weight of *C. zofingiensis* can reach up to 98.4 g L^−1^ in fermentation, which is much higher than that under photoautotrophic conditions (Zhang et al., 2017). However, the astaxanthin content in *C. zofingiensis* heterotrophic cells is about 10-fold lower than that of the photoautotrophic cells, of which the former is 0.06% of DW and the latter is 0.5% of DW (Zhang et al., 2016).

Numerous studies have demonstrated the biotechnical significance of heterotrophic cultivation for microalgal biomass and chemicals production (Lee Chang et al., 2015; Morales-Sánchez et al., 2015; Hu et al., 2018). Through optimizing the components of growth media and substrate feeding strategy, ultra-high biomass yield (>200 g L^−1^) can be achieved for several production strains such as *Chlorella sorokiniana* (Jin et al., 2021), *Chlorella sorokiniana* strain CMBB276 (Xu et al., 2021) and *Scenedesmus acuminatus* (Jin et al., 2020). The capabilities of these species in achieving such high biomass yield have been attributable to the highly active starch biosynthesis under heterotrophic conditions, considering the carbon substrate (i.e. glucose) can be directly utilized as the building blocks of starch, which eliminates the loss of carbon atoms in central carbon metabolism (e.g. from pyruvate to acetyl-CoA) and for reducing equivalents production. Thus, it remains disputed whether microalgae with either high protein or lipid contents possess the potential to reach biomass and products yield as high as that of those starch-producing strains.

For *C. zofingiensis*, challenges in establishing ultra-high cell density heterotrophic cultivation technology are more complicated than that for the other oleaginous microalgae, since biosynthesis astaxanthin needs to consume large amounts of oxygen as indicated by previous study in *H. pluvialis* (Li et al., 2008). Moreover, understanding about astaxanthin accumulation under heterotrophic conditions is limited. Accumulation of astaxanthin has been considered as a protective response triggered by photo-oxidative stresses, which is linked to over-reduction of photosynthetic electron transport chain and production of reactive oxygen species in microalgal cells. Thus, astaxanthin can be induced by high-light, nutrient depletion and high-salinity stresses in *H. pluvialis* and many other microalgae under photoautotrophic conditions. In contrast, it is unlikely to impose the photo-oxidative stresses on *C. zofingiensis* cells grown under heterotrophic conditions. Thus, it remains to be explored how to trigger the biosynthesis of the secondary carotenoid-astaxanthin. It was found that the high C/N ratio can enhance the astaxanthin production in *C. zofingiensis* in dark (Ip and Chen, 2005). On the other hand, phytohormones as chemical messengers can regulate plant and microalgae growth, development, metabolism, as well as environmental stress responses (Voß et al., 2014; Lu and Xu, 2015). Several previous studies suggested that various phytohormones, such as cytokinins, ethylene precursor, gibberellic acid, and auxins can enhance the accumulation of biomass and metabolites, such as pigments, proteins, carbohydrates, and lipids in microalgae (Hunt et al., 2010; Gao et al., 2013; Bajguz and Piotrowska-Niczyporuk, 2014; Jiang et al., 2015).

This present work aimed to establish a two-stage cultivation strategy to enhance astaxanthin production of *C. zofingiensis* under heterotrophic conditions. Firstly, the growth conditions including nitrogen source, temperature, pH, and substrate concentration were optimized in the bench-top fermentor, and the optimal conditions were scaled-up at the pilot scale. To induce the astaxanthin biosynthesis, the effects of various of phytohormones were compared, from which the most effective phytohormone combined with high C/N and salinity stresses were introduced to the high-cell density heterotrophic conditions. The results of this study suggested heterotrophic cultivation of *C. zofingiensis* is a competitive technical route for producing astaxanthin.

## Materials and Methods

### Microalgal Strain and Growth Media

*Chromochloris zofingiensis* (ATCC 30412) was obtained from the American Type Culture Collection (ATCC, Rockville, United States) and was maintained in the modified Endo growth medium consisting of 3 g L^−1^ KNO_3_, with the addition of 15 g L^−1^ agar for plates. The modified Endo growth medium was adjusted to pH 6.5 with 3M NaOH before autoclaving at 121 °C for 15 min.

### Culture Conditions

A single colony of *C. zofingiensis* was transferred in 250 ml Erlenmeyer flasks containing 100 ml sterilized modified Endo growth medium and cultivated for 7 days at 26°C with orbital shaking at 180 rpm. Microalgal cells in the exponential phase were used as the inoculum at 10% (v/v) for fermentation experiments. Bench fermentation experiments were carried out in the 7.5 L BIOFLO and CELLIGEN 310(New Brunswick. Scientific Co., Edison, NJ) fermenters with an initial working volume of 3 L. The fermentor was continuously aerated through a 0.2 μm membrane filter at a flow rate of 2.0 L min^−1^. To minimize evaporation, the vent gas passed through a modified condenser mounted on the head plate of the fermentor. Dissolved oxygen (DO) was controlled automatically at about 20% by coupling with the stirring speed. The changes of DO and stirring speeding during the heterotrophic cultivation period are shown in Supplementary Figure S1. The pH was maintained at 6.5 ± 0.5 by the automatic addition of NH_3_·H_2_O. For the heterotrophic cultivation of *C. zofingiensis*, 20 g·L^−1^glucose was initially added to the fermentation medium. When the glucose concentration decreased to 5 g L^−1^, fed-batch fermentation was carried out by continuously feeding the cell cultures with a 25-fold concentrated batch medium containing 750 g·L^−1^ glucose at a predetermined flow rate using a peristaltic pump (Cole Parmer, Vernon Hills, IL, United States) and maintained the range of glucose at a stable level (e.g., 0–30 g L^−1^). The maximum culture volume was 5L, from which 500 ml of the culture was discharged when the culture volume almost reached the maximum. The morphology of microalgal cells was observed with an Olympus BX-53 microscope (Olympus Optical Co. Ltd., Tokyo, Japan).

For the heterotrophic cultivation in the 7.5 L fermenters, the initial nitrogen source in the modified Endo growth medium was replaced by sodium nitrate (NaNO_3_), urea ((NH_2_)_2_CO), and ammonium chloride (NH_4_Cl), respectively. For these heterotrophic culturing media, the molar ratio of the nitrogen to carbon source was maintained at 32:1, respectively. The pH values were controlled by feeding with NH_3_·H_2_O or 3M H_3_PO_4_ solutions.

**FIGURE 1:**
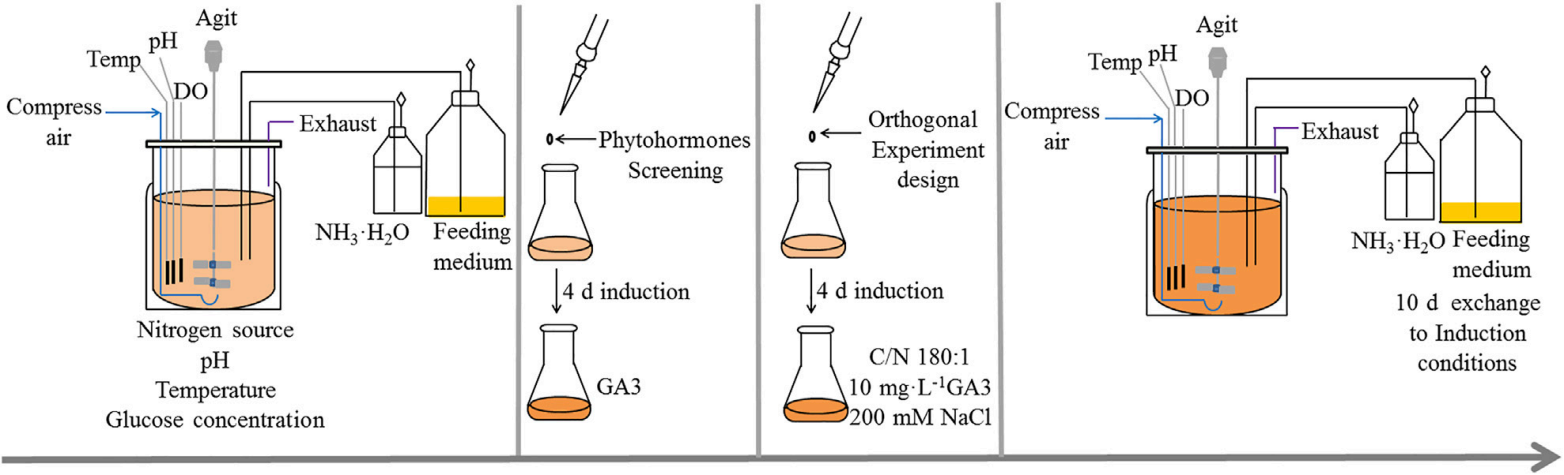
Experimental procedures for *C. zofingiensis* produce astaxanthin under heterotrophic conditions. The algae (with initial density of 1–1.5 g L^−1^) kept growing for 10 days with the optimal heterotrophic conditions. The phytohormones screening and orthogonal experiment were conducted in Erlenmeyer flasks to find the optimal condition for astaxanthin accumulation in *C. zofingiensis.* The induction conditions were verified in 7.5 L fermentors.

The experimental procedures are schematically illustrated in Figure 1.

To mitigate the chance of bacterial contamination, the sterile operation procedure was strictly followed, which included using the ultraviolet lamp frequently to sterilize the air in the culture room, autoclaving, the culture media and fermentors at 121°Cfor 30 min, and conducting sterility test before inoculation. In addition, air filters were installed at the air inlet of the fermentors and were frequently changed to keep the air flowing into the fermentors clean.

### Measurement of Biomass dry Weight and Glucose Consumption

The microalgal biomass dry weight was determined as previously reported (Mao et al., 2020). In brief, samples at each time point were harvested and washed twice with 0.5M NH_4_HCO_3_ and filtered through a pre-dried and pre-weighed dry GF/C filter paper of 1.2 μm pore size (Whatman, Life Sciences, United Kingdom), which was then dried at 105°C in a vacuum oven overnight. The Whatman GF/C filter papers were placed in a desiccator for 20 min to allow the temperature cooling down before dry weight measurement. The glucose concentration was monitored by using a Gold-Accu Glucose Monitoring System (Model BGMS-1; Sinocare Inc., Changsha, China).

### Phytohormones Screening

Two-stage cultivation lasting for 14 days was performed to screen among phytohormones including GA3, Indole-3-acetic acid (IAA), Indole-3-propionic (IPA), Indole-3-butyric acid (IBA), 1-Naphthylacetic acid (NAA), and 1-aminocyclopropane-1-carboxylic acid (ACC) that can enhance astaxanthin accumulation (Aladdin Chemical Reagent Co., Ltd, Shanghai). Their physic-chemical properties are presented in Supplementary Table S1. In the first stage, algal cells were grown in 7.5 L fermenters to reach the biomass yield of 180 g L^−1^ during 10 days. Then the collected algal cells were inoculated into the Erlenmeyer flasks to maintain the initial cell density of algae suspension at 2–3 g L^−1^. Stock solutions of phytohormones were prepared in sterile water or ethanol and then directly added into the heterotrophic culture condition in the Erlenmeyer flasks at the designated concentration.

### Orthogonal Experiment Design

The induction conditions were optimized by using an L_16_ orthogonal array. The C/N ratio, GA3 concentration, and NaCl concentration were chosen as the conditional factors of the orthogonal experiments. Each factor was set at four levels as shown in Supplementary Table S2. The pH in each treatment group was successfully controlled in the range of 6.5–7. The inoculum concentrations and heterotrophic culture condition were performed using the same as aforementioned conditions in the Erlenmeyer flasks. The optimal induction condition obtained through orthogonal experiments was verified during the induction period in the 7.5 L fermenters.

### Pigments Analysis

The microalgal cells were harvested by centrifugation at 3,000 *g* for 5 min and washed twice in PBS buffer solution. The cell pellets were freeze-dried to completely remove water. Pigments were extracted by using methanol/dichloromethane (3:1, v/v) till the residues became colorless. The extracts were centrifuged at 12,000 × g for 5 min to remove the debris, and the supernatants were dried, recovered in 0.5 ml of extraction solution, and filtrated through 0.22 μm membrane filters (Pall Life Science, United States) before High-Performance Liquid Chromatography (HPLC) analysis. The pigments were separated on a Waters Symmetry C_18_ (150 × 4.6 mm, 5 μm) column at 35°C. Ten μL aliquot of the sample was injected into the waters e2695 HPLC (Waters Associates, Milford, MA, United States) system equipped with a 2998 photodiode array detector (Waters, Milford, MA, United States). The mobile phase consisted of eluent A (dichloromethane/methanol/acetonitrile/water, 5.0:85.0:5.5:4.5, v/v) and eluent B (dichloromethane/methanol/acetonitrile/water, 25.0:28.0:42.5:4.5, v/v). Pigments were separated by using the following gradient procedure: 0% B for 8 min, then increasing the gradient to 100% of B within 6 min and holding for 40 min. The flow rate was 1.0 ml min^−1^. Astaxanthin standard (Sigma-Aldrich, St. Louis, MO, United States) was used as a calibrant for quantification.

### Total Fatty Acid Content Quantification

Approximately 10 mg of lyophilized microalgal powder was weighed into a 1.5 ml GC vial, which was used for total fatty acid analysis. Two hundreds microliters of chloroform: methanol (2:1, v/v), 25 μL of ^13^C:0 (200 μg mL^−1^), and 300 μL of 5% HCl: methanol were added, respectively. The mixture samples were heated at 85 °C for 1 h. After cooling down to room temperature, 1 ml hexane was mixed with the in the room temperature for 1–4 h. Nine hundreds microliters of hexane and 1-μL of hexane top layer were transferred into GC vials and then mixed with 25 μL pentadecane standard (200 μg mL^−1^) for fatty acid methyl esters (FAME) analysis, which was performed by using an Agilent 7890B gas chromatography equipped with 5977A mass spectrometry (GC-MS). The chromatographic separation was using the same conditions as previously described (Wu et al., 2019).

### Statistical Analysis

All the experiments were conducted in at least triplicate. Experimental results shown in the figures are expressed as the mean value ±SD. The data were analyzed by one-way analysis of variance using Dunnett multiple comparison tests and GraphPad Prism (v. 7.0 for Windows; GraphPad Software, Inc., CA, United States). A value of *p* < 0.05 was considered statistically significant.

## Results

### Optimization of Heterotrophic Culturing Conditions for *C. zofingiensis*

**FIGURE 2:**
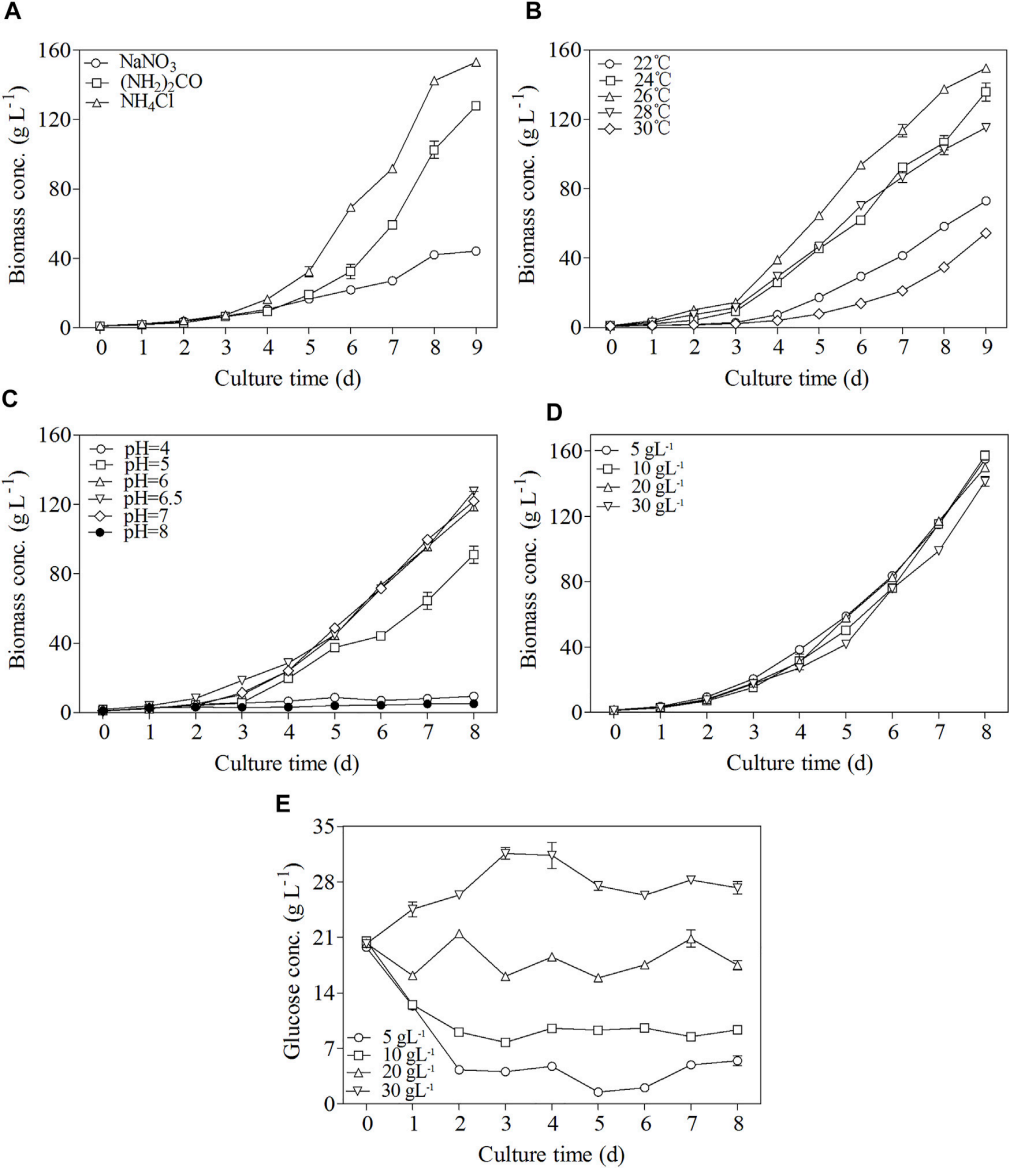
Effects of different nitrogen sources **(A)**, temperature **(B)**, pH **(C)**, glucose concentration **(D)**, and change of glucose concentration **(E)** on *C. zofingiensis* cellular growth under heterotrophic conditions in 7.5 L fermentors. Data are mean values ±SD from triplicate measurements (n = 3). All cultures were performed in 7.5 L fermentors using NH_4_Cl as the nitrogen source.

The effects of various nitrogen sources including NaNO_3_ (NH_2_)_2_CO, and NH_4_Cl on the growth of *C. zofingiensis* were investigated in 7.5 L fermenters by using fed-batch culture mode. As shown in Figure 2A, *C. zofingiensis* cell can utilize NaNO_3_ (NH_2_)_2_CO, and NH_4_Cl as nitrogen source, among which NH_4_Cl and urea sustained rapid microalgal growth. Notably, an ultrahigh biomass concentration of 153 g L^−1^ was achieved in the modified Endo culture medium containing NH_4_Cl as the nitrogen source. Therefore, NH_4_Cl was selected as the nitrogen source suitable for *C. zofingiensis* growth and was used in the other optimization experiments.

Within the temperature range of 24–28°C, *C. zofingiensis* showed a rapid growth trend, and the highest biomass concentration of 149.5 g L^−1^ was achieved at 26°C after 9 days of cultivation (Figure 2B). However, further increases in the cultivation temperature can inhibit the growth of microalgal cells (Figure 2B). Thus, 26°C was chosen as the optimal culture temperature.

The growth parameters of *C. zofingiensis* at different culture pH values are shown in Figure 2C. The culture pH was controlled at 4.0–8.0 via utilizing the pH-stat mode. At pH 6.5, the highest biomass concentration of 127.7 g L^−1^ was achieved at the end of the 8-day fermentation.

Glucose is a common organic carbon for the heterotrophic cultivation of microalgae. The effects of glucose concentration on *C. zofingiensis* growth under heterotrophic conditions are shown in Figure 2D,E. The glucose concentration in the fermentation medium was continuously measured to control the glucose in the medium maintained at a stable level (Figure 2E). When the glucose in the fermentation medium was consumed to the concentration of approximately 5 g L^−1^, additional media with glucose were fed to the cultures to reach the initial glucose concentration. The results exhibited that the biomass concentrations were very close among all the microalgal cell cultures with different glucose concentrations. The highest biomass concentration reached 157.3 g L^−1^ after 8 days of cultivation when the glucose concentrations were maintained at 10 g L^−1^.

### Pilot-Scale Heterotrophic Culture of *C. zofingiensis* Under the Optimal Culture Conditions

**FIGURE 3:**
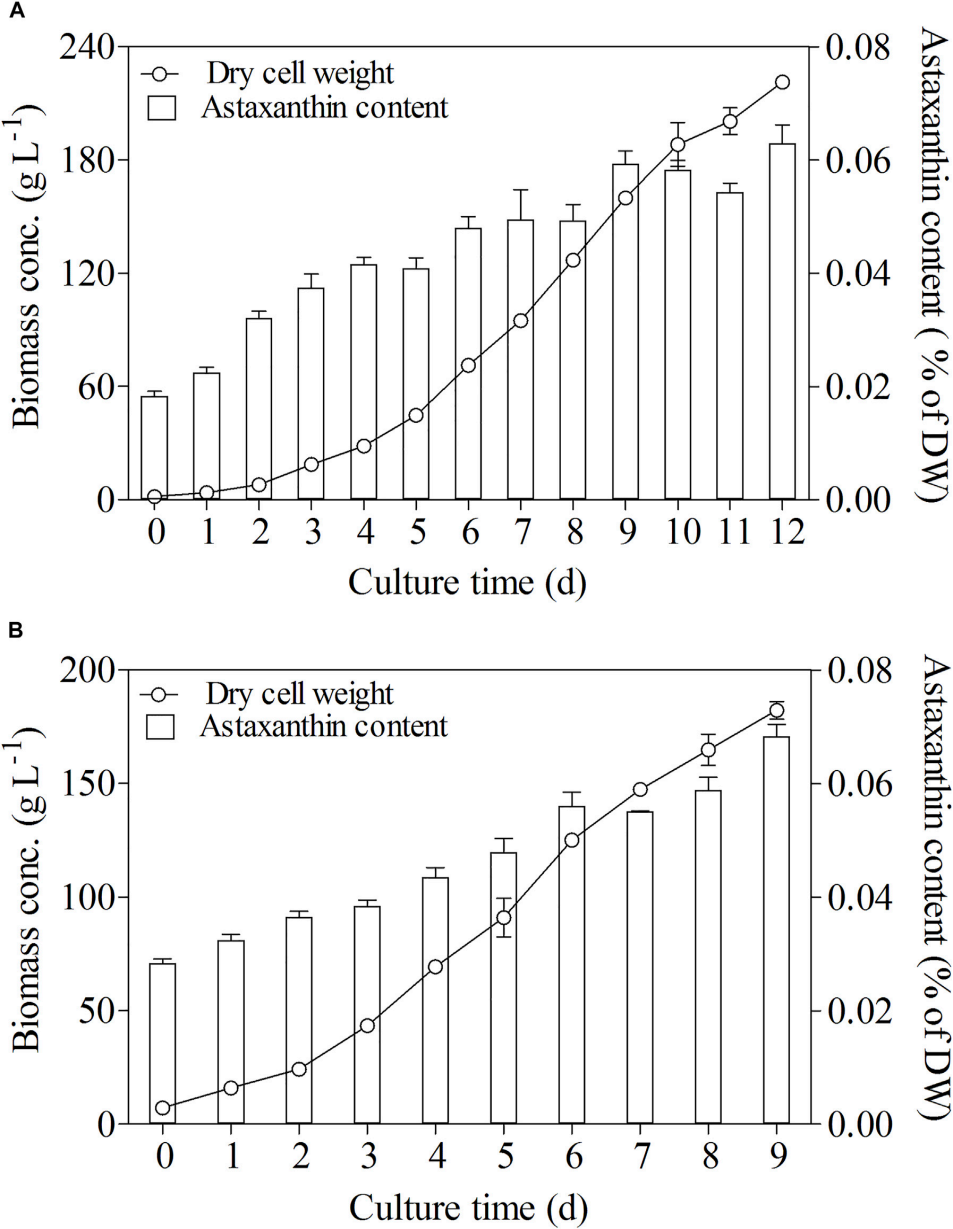
Biomass and astaxanthin content in fed-batch fermentation of *C. zofingiensis* in 7.5 L fermentors **(A)** and 500 L fermentors **(B)** with optimal cultural conditions. Data are mean values ±SD from triplicate measurements (n = 3).

Glucose was the best carbon and energy source for heterotrophic growth of *C. zofingiensis* which showed ultrahigh biomass concentrations. Heterotrophic cultures with glucose not only enhanced biomass concentration but also stimulated astaxanthin accumulation (Zhang et al., 2019). The astaxanthin content in *C. zofingiensis* increased considerably with increasing biomass concentration in the heterotrophic fed-batch cultures (Figure 3). When the optimal conditions were adopted for the scaling-up experiment conducted in a 500 L fermentor, the highest biomass concentration reached 182.3 g L^−1^ on Day 9, which was 14% higher than the highest cell density (160 g L^−1^) achieved in the 7.5 L fermentor. As compared with the 7.5 L fermentor, the significant enhancement of *C. zofingiensis* biomass in the 500 L fermentor revealed that *C. zofingiensis* has the potential for large-scale industrial production. Meanwhile, the maximum astaxanthin content in 500 L reached 0.068% of DW, slightly higher than the highest astaxanthin content (0.063%) attained in 7.5 L fermentor.

### Screening Phytohormones That can Efficiently Enhance Astaxanthin Production in *C. zofingiensis*

The effects of a variety of phytohormones on the astaxanthin production of *C. zofingiensis* were investigated. As shown in Table 1, GA3, IAA, IPA, IBA, NAA, and ACC all demonstrated significant effects on *C. zofingiensis* growth characteristics, as compared to the control (*p* < 0.01). Specifically, GA3, IAA, IPA, NAA, and ACC enhanced astaxanthin content, whereas inhibited *C. zofingiensis* biomass accumulation and glucose assimilation (Table 1).

**TABLE 1:** *C. zofingiensis* biomass concentration (g·L^−1^), pH, glucose concentration (g·L^−1^), and astaxanthin content (% of DW) at the termination of each experiment.

Phytohormones concentration	Biomass concentration (g·L^−1^)	pH	Glucose concentration (g·L^−1^)	Astaxanthin content (% of DW)
Control	6.73 ± 0.247	6.02 ± 0.057	9.40 ± 0.085	0.062 ± 0.002
GA				
5 mg L^−1^	4.70 ± 0.144	5.60 ± 0.028	25.29 ± 0.127	0.080 ± 0.002
10 mg L^−1^	5.25 ± 0.212	5.54 ± 0.007	27.07 ± 0.099	0.080 ± 0.002
25 mg L^−1^	4.35 ± 0.071	5.44 ± 0.049	27.75 ± 0.212	0.081 ± 0.003
50 mg L^−1^	3.75 ± 0.212	5.59 ± 0.014	27.99 ± 0.381	0.101 ± 0.003
IAA				
15 mg L^−1^	6.25 ± 0.354	5.55 ± 0.071	20.90 ± 1.273	0.089 ± 0.001
70 mg L^−1^	4.80 ± 0.141	5.40 ± 0.141	23.89 ± 0.156	0.096 ± 0.002
150 mg L^−1^	4.75 ± 0.071	5.42 ± 0.049	25.90 ± 0.141	0.094 ± 0.005
250 mg L^−1^	4.55 ± 0.072	5.24 ± 0.085	26.97 ± 0.764	0.076 ± 0.005
IPA				
10 mg L^−1^	5.85 ± 0.071	5.70 ± 0.091	20.93 ± 1.315	0.083 ± 0.001
60 mg L^−1^	5.10 ± 0.283	5.66 ± 0.042	23.45 ± 0.700	0.089 ± 0.005
120 mg L^−1^	4.70 ± 0.141	5.60 ± 0.007	25.38 ± 0.509	0.097 ± 0.002
240 mg L^−1^	3.85 ± 0.071	5.51 ± 0.014	27.22 ± 0.311	0.086 ± 0.002
IBA				
10 mg L^−1^	5.31 ± 0.233	5.80 ± 0.064	23.80 ± 0.304	0.075 ± 0.003
50 mg L^−1^	4.61 ± 0.234	5.71 ± 0.071	24.45 ± 0.212	0.060 ± 0.002
90 mg L^−1^	3.93 ± 0.035	5.72 ± 0.028	25.53 ± 0.177	0.059 ± 0.004
130 mg L^−1^	3.87 ± 0.099	5.68 ± 0.035	25.27 ± 0.191	0.057 ± 0.003
NAA				
10 mg L^−1^	3.65 ± 0.071	5.66 ± 0.021	25.81 ± 0.155	0.082 ± 0.005
20 mg L^−1^	3.35 ± 0.071	5.55 ± 0.063	27.00 ± 0.156	0.078 ± 0.002
40 mg L^−1^	3.18 ± 0.035	5.35 ± 0.070	27.92 ± 0.106	0.039 ± 0.001
60 mg L^−1^	3.05 ± 0.070	5.69 ± 0.014	28.10 ± 0.212	0.036 ± 0.003
ACC				
5 mg L^−1^	5.66 ± 0.198	5.77 ± 0.021	27.11 ± 0.127	0.064 ± 0.002
10 mg L^−1^	5.81 ± 0.106	5.54 ± 0.020	26.68 ± 0.057	0.068 ± 0.003
20 mg L^−1^	4.76 ± 0.148	5.43 ± 0.042	24.63 ± 0.240	0.066 ± 0.002
40 mg L^−1^	4.31 ± 0.240	5.34 ± 0.021	25.70 ± 0.141	0.068 ± 0.001

Table 1 demonstrated that GA3 was the most effective phytohormone to enhance astaxanthin content, which was 50.7% higher than that of the control (*p* < 0.001). Similarly, IAA and IPA significantly increased astaxanthin content to 0.096 and 0.097% of DW, respectively, significantly higher than that of the control (*p* < 0.001). In addition, low concentration of IBA and NAA could stimulate astaxanthin accumulation, whereas these phytochromes might inhibit astaxanthin accumulation at high concentrations. The addition of ACC showed no significant effect on astaxanthin accumulation as compared to the control.

The inhibition effect of phytohormone on biomass accumulation and glucose assimilation was enhanced with increasing phytohormone concentrations (Table 1). With the use of phytohormone, the pH value of culture decreased constantly from the initial value of 6.1 (*p* < 0.01). However, the enhanced astaxanthin production was accompanied by the reduced biomass yield. The consumption of glucose by *C. zofingiensis* was significantly inhibited when the phytochromes were added as compared to the control (*p* < 0.05).

### Optimization of the Induction Conditions

**FIGURE 4:**
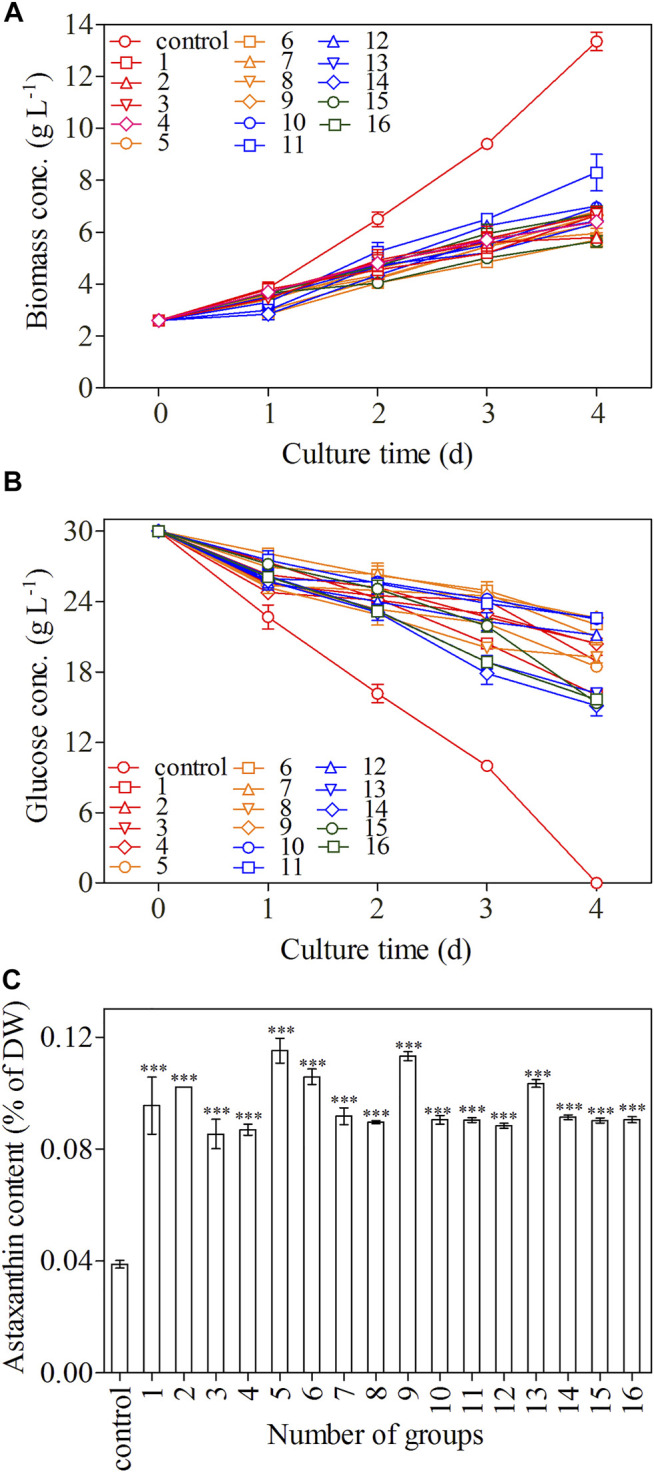
Biomass concentration **(A)**, glucose concentration **(B)**, and astaxanthin content **(C)** of *C. zofingiensis* in different treatment groups. The biomass concentration, glucose concentration, and astaxanthin content were monitored daily during 4-day induction; astaxanthin content was measured on day 4. All cultures were conducted in Erlenmeyer flasks with modified Endo culture medium containing NH_4_Cl as the nitrogen source. Data are mean values ±SD from triplicate measurements (n = 3) (See Table 2 for detailed information on treatment groups 1–16).

To further enhance the astaxanthin production in *C. zofingiensis*, algal cells were induced with the addition of GA3 combined with simultaneously increasing C/N and salinity. The conditions were optimized with orthogonal experiments. The astaxanthin contents in all treatment groups were greatly enhanced as compared to the control (*p* < 0.001, Figure 4C). According to the results, the C/N 180:1, 10 mg L^−1^ GA3, and 200 mM NaCl turned out to be the most effective treatment group to promote astaxanthin production, in which the astaxanthin content reached 0.115% of DW. The inducing effect of the C/N 220:1, 10 mg L^−1^ GA3, and 400 mM NaCl on the astaxanthin production is very close to that of the previous one, which was higher than the other treatment groups.

The growth trends of *C. zofingiensis* in terms of biomass accumulation were similar among different treatments during the 4-day induction period and were inhibited as compared to the control (*p* < 0.01, Figure 4A). *C. zofingiensis* exhibited a short adaptation period in each treatment group (Figure 4A). The biomass concentration obtained under the conditions of C/N 220:1, 100 mg L^−1^ GA3, and 100 mM NaCl was the highest, whereas that obtained under the conditions of the C/N 280:1, 100 mg L^−1^ GA3, and 200 mM NaCl was the lowest. The consumption of glucose was similar among different treatments and was slower than that of the control (Figure 4B). Among the 16 treatment groups, the C/N 280:1, 50 mg L^−1^ GA3, and 400 mM NaCl showed the fastest consumption of glucose during the induction period, whereas C/N 220:1, 100 mg L^−1^ GA3, and 100 mM NaCl showed the slowest glucose assimilation.

**FIGURE 5:**
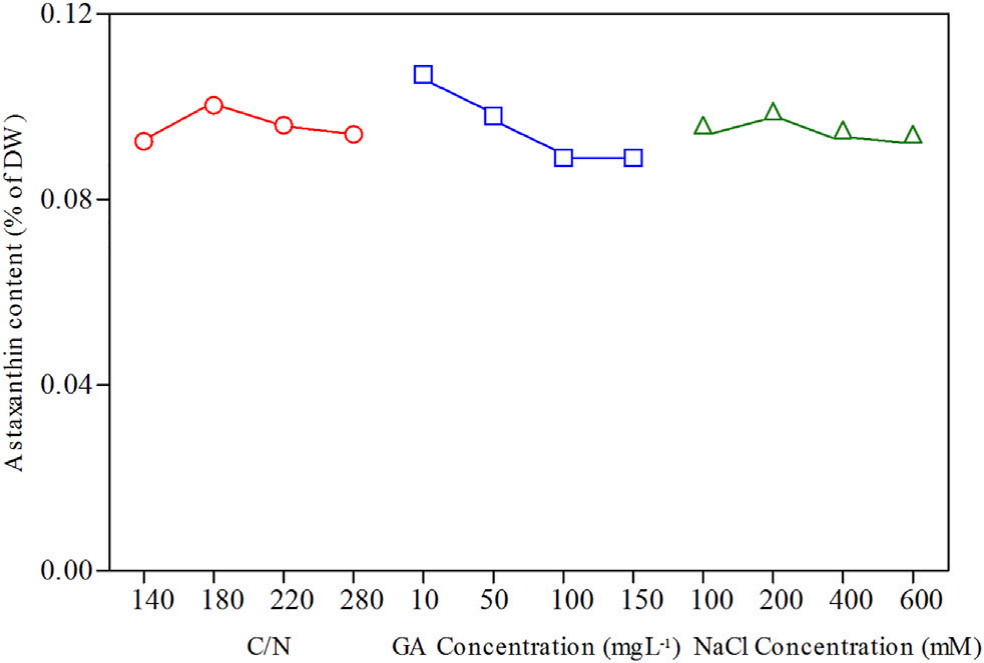
Orthogonal experimental effect curves for astaxanthin content of *C. zofingiensis.* All treatment groups were performed in Erlenmeyer flasks.

According to Figure 5, the optimal conditions for astaxanthin accumulation were C/N 180:1, 10 mg L^−1^ GA3, and 200 mM NaCl concentration. The orthogonal test ANOVA results are presented in Table 2. In addition, based on the orthogonal experiment results, it can be concluded that GA3 concentration exerts the most stimulatory effects on the accumulation of astaxanthin among the three factors.

**TABLE 2:** Orthogonal range analysis and variance analysis.

No	A: C/N ratio	B: GA3 (mg·L^−1^)	C:NaCl (mM)	Astaxanthin content (% of DW)
1	140:1	10	100	0.096
2	140:1	50	200	0.102
3	140:1	100	400	0.085
4	140:1	150	600	0.087
5	180:1	10	200	0.115
6	180:1	50	100	0.106
7	180:1	100	600	0.092
8	180:1	150	400	0.090
9	220:1	10	400	0.113
10	220:1	50	600	0.091
11	220:1	100	100	0.090
12	220:1	150	200	0.088
13	280:1	10	600	0.104
14	280:1	50	400	0.091
15	280:1	100	200	0.090
16	280:1	150	100	0.091
K1	0.370	0.428	0.383	∑1.531
K2	0.403	0.390	0.395	—
K3	0.382	0.357	0.379	—
K4	0.376	0.356	0.374	—
k1	0.093	0.107	0.096	—
k2	0.100	0.098	0.099	—
k3	0.096	0.089	0.095	—
k4	0.094	0.089	0.094	—
R	0.007	0.018	0.003	—
F*	1.151	7.045	0.523	—
Significant	—	*	—	—
Optimal case	—	A_2_B_1_C_2_	—	—

K1, K2, and K3 are the sum of each level value of each factor.

k1, k2, and k3 are the average of each level value of each factor.

R is the range of each factor.

### Two-Stage Heterotrophic Cultivation of *C. zofingiensis* for Astaxanthin Production in 7.5 L Fermenters

**FIGURE 6:**
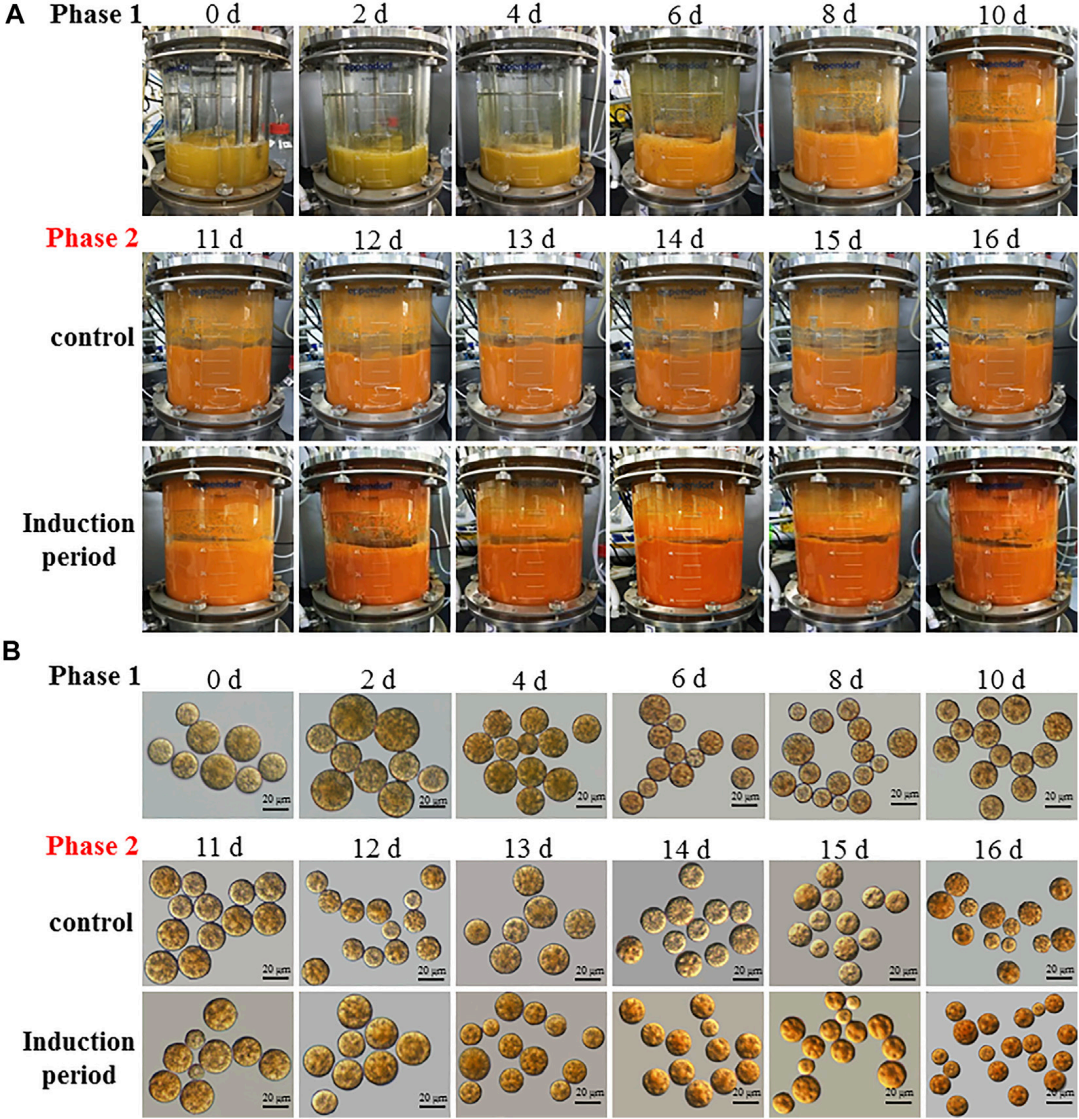
The culture appearance **(A)** and morphological **(B)** changes of *C. zofingiensis* cells during the induction period in the 7.5 L fermentors. Induction conditions, the C/N ratio of the feeding medium change to 180:1 with the addition of 10 mg L^−1^ GA3 and 200 mM NaCl at the end of 10 days.

A two-stage process with the combination of the optimal induction conditions (i.e. C/N 180:1, 10 mg L^−1^ GA3 and 200 mM NaCl) with the high cell-density cultivation conditions was employed for the heterotrophic cultivation of *C. zofingiensis* in the 7.5L fermentor. The changes in the appearance of *C. zofingiensis* cell culture and the cell morphology were shown in Figure 6. The color of the algal cell culture gradually turn to reddish from green over 16 days (Figure 6A). Once the algal cells were subjected to the induction conditions (stage II), the changes in the color are more obvious and rapid than that of the control. Significant decreases in the size of *C. zofingiensis* cells were observed during the induction period, as compared with that of the control (Figure 6B).

**FIGURE 7:**
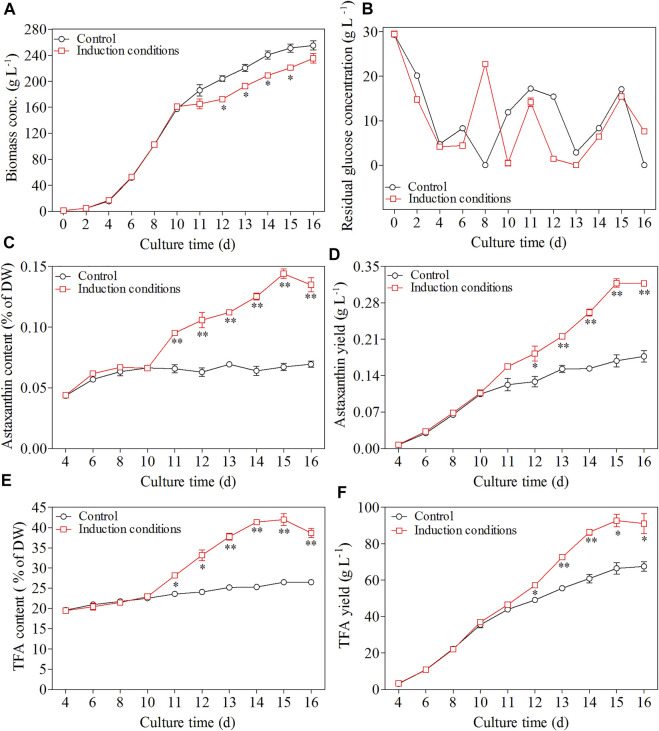
Effect of induction conditions on the growth of *C. zofingiensis* and astaxanthin production during the heterotrophic cultivation in 7.5 L fermentors. **(A)** Time course of dry cell weight. **(B)** Residual glucose concentration. **(C)** Astaxanthin content. **(D)** Astaxanthin yield. **(E)** TFA content **(F)** TFA yield. Induction conditions, the C/N ratio of the feeding medium change to 180:1 with the addition of 10 mg L−1 GA3 and 200 mM NaCl at the end of 10 days. Data are mean values ±SD from triplicate measurements (n = 3). * and ** were statistically significant at *p* < 0.05 and *p* < 0.01, respectively.

In the first phase, the dry cell weight of *C. zofingiensis* increased dramatically and reached 160 g L^−1^ after 10 days of cultivation. After Day 10, when the C/N ratio of the feeding medium changed to 180:1, and GA3 and NaCl were added at concentrations of 10 mg L^−1^ and 200 mM, respectively, the microalgal cells exhibited a short adaption during the first 2 days of the induction stage, and then the dry cell weight of *C. zofingiensis* remained at a stable level. Afterwards, the dry cell weight of *C. zofingiensis* increased rapidly and reached the highest cell density at 235.4 g L^−1^ on Day 16 (Figure 7A). Figure 7B demonstrated that *C. zofingiensis* could adapt to a wide range of glucose concentrations.

The changes in astaxanthin content and yield during the whole cultivation period of *C. zofingiensis* were shown in Figure 7C,D. In the first stage, astaxanthin content and yield increased with cultivation time. Notably, the astaxanthin content and yield reached 0.066% of DW and 0.107 g L^−1^, respectively, after 10 days of cultivation. From the beginning of the second stage, the astaxanthin content and yield increased more dramatically as compared to that of the control (*p* < 0.05). The highest astaxanthin content and yield were achieved at 0.144% of DW and 0.318 g L^−1^, respectively, after 5 days of induction.

*C. zofingiensis* cells not only possess the ability to produce astaxanthin but also accumulate lipids under induction conditions. Thus, the TFA contents were analyzed in this study as well. As shown in Figure 7E, the TFA content increased from 23 to 42% of DW during 5-day induction, and then slightly declined to 38.6% of DW on Day 16, which was still 56% higher than that of the control. Under the two-stage cultivation conditions, the maximum TFA yield of 92.7 g L^−1^ was achieved on Day 15 (the fifth day after induction) and then leveled off, which was significantly higher than that of the control (66.5 g L^−1^, Figure 7F). These results indicate the optimized induction conditions can simultaneously stimulate lipid and astaxanthin production.

## Discussion

In this study, a two-stage heterotrophic cultivation process was developed for *C. zofingiensis*, which enabled achieving the highest biomass yield and astaxanthin content to date. In the first stage when the *C. zofingiensis* cells were grown under the optimal conditions, the maximum biomass yield of 221.3 g L^−1^ and 182.3 g L^−1^ was attained in 7.5-L (on Day 12) and 500-L fed-batch fermenters (on Day 9), respectively. During the second stage, the astaxanthin was substantially accumulated through employing the inductive conditions that combined the phytochrome GA3, high C/N, and salinity. At the endpoint of fermentation (Day 16), the astaxanthin yield reached 0.318 g L^−1^ when biomass yield was 235.4 g L^−1^ and astaxanthin content was 0.144% of DW, which was 5.4-fold higher than the highest record that had been reported (Sun et al., 2008). Such high astaxanthin yield surpassed the level of *H. pluvialis*, of which the astaxanthin yield is about 0.077 g L^−1^ (López et al., 2006).

The optimal growth conditions attained in this study distinguished *C. zofingiensis* from *C. sorokiniana* and *S. acuminatus*, the two green algae that can be grown under heterotrophic conditions to ultrahigh cell densities as well. Firstly, *C. zofinginensis* favored relatively lower temperature (i.e. 26°C) as compared to *C. sorokiniana* and *S. acuminatus*, for which the optimal temperatures were both above 30°C. Secondly, little difference in the cell growth was observed among the groups with different glucose concentrations, suggesting *C. zofingiensis* can adapt to a wide range of glucose concentrations (Figure 2D). In contrast, glucose concentration exerted significant impacts on the growth of *C. sorokiniana* and *S. acuminatus*. In addition, when *C. zofingiensis* was grown under neutral condition (pH = 7), it grew as fast as that under weak acidic conditions, though it is usually believed that heterotrophic microalgae prefer to the weak acidic conditions since microalgal cells transport one molecule of proton in stoichiometry for uptake of one molecule of sugar from the growth media (Perez-Garcia et al., 2011). It is noteworthy the conversion efficiency of the glucose to biomass is 50% for *C. zofingiensis* grown under favorable conditions, which is lower than that of *C. sorokiniana* and *S. acuminatus* (i.e. 60%). The relative low glucose conversion efficiency for *C. zofingiensis* is attributable to its higher lipid contents than that of *C. sorokiniana* and *S. acuminatus.*

Although heterotrophic cultivation of *C. zofingiensis* is regarded as a potential technical route for natural astaxanthin production, the content of astaxanthin obtained under such mode is generally lower than that obtained under photoautotrophic modes. In this study, GA3 is proved to be the most effective factor to promote astaxanthin biosynthesis in heterotrophically-grown *C. zofingiensis*. GA3 was found to induce astaxanthin biosynthesis in *H. pluvialis* through up-regulating the transcription of β-carotene ketolase genes, which is involved in converting β-carotene to the canxathathin-the precursor of astaxanthin (Lu et al., 2010; Yu et al., 2015). GA is a diterpene phytochrome that control the growth and development of plants, including seed germination, hypocotyl elongation, flower initaiation, stem elongation via enhanced cell division and elongation. Thus, GA3 may promote the cell devision of *C. zofingiensis* under heterotrophic conditions. GA can cause rewiring of central carbon metabolism during fruit setting in tomatoes, including increasing the supply of hexoses, hexose phosphates, the intermediates and derivatives of glycolysis, and tricarboxylic acid, and these responses are suggested to be predominantly under transcriptional control (Shinozaki et al., 2020). These findings can explain why GA3 can promote astaxanthin biosynthesis under both photoautotrophy and heterotrophy conditions in a photooxidative signals-independent manner. However, increasing C/N might not be specific enough, since large amounts of carbon were rechanneled into the fatty acid biosynthesis (Figure 7E). As a result, the glucose conversion efficiency during the second stage was 36%, much lower than that under favorable conditions. In future studies, it is necessary to design strategies that can specifically increase the supply of precursors for astaxanthin biosynthesis.

## Conclusion

In this study, a two-stage heterotrophic cultivation process that enabled achievement of both high biomass and astaxanthin yield was developed for *C. zofingiensis*. The fed-batch heterotrophic cultivation was optimized in 7.5 L fermentor and scaled-up in 500 L fermentor. The phytochrome GA3 was selected as one of the most effective phytohormones to induce astaxanthin accumulation, and it was combined with high C/N ratio and NaCl concentration to enhance axtaxanthin production in fed-batch fermentation. By employing the novel two-stage heterotrophic cultivation strategy, the astaxathin yield from *C. zofingiensis* surpassed that of *H. pluvialis*, indicating it is a promising technical route to sustainablly produce astaxanthin under the well-controlled conditions.

## Data Availability

The original contributions presented in the study are included in the article/Supplementary Material, further inquiries can be directed to the corresponding author.
