# Supplementary material for: Effective Two-Stage Heterotrophic Cultivation of the Unicellular Green Microalga Chromochloris zofingiensis Enabled Ultrahigh Biomass and Astaxanthin Production
Source: Front Bioeng Biotechnol. 2022 Feb 24;10:834230. doi: 10.3389/fbioe.2022.834230 (PMC8907917; doi:10.3389/fbioe.2022.834230)
Supplement: Supplementary file 1 [file DataSheet1.docx]

**Effective two-stage heterotrophic cultivation of the unicellular green microalga *Chromochloris zofingiensis* enabled ultrahigh biomass and astaxanthin production**

Qiaohong Chen^1, 4, §^, Yi Chen^1, 4, §^, Quan Xu^1^, Hu Jin^1^, Qiang Hu^2^, and Danxiang Han^1,3*^

^1^ Center for Microalgal Biotechnology and Biofuels, Institute of Hydrobiology, Chinese Academy of Sciences, Wuhan 430072, China.

^2^ Institute for Advanced Study, Shenzhen University, Shenzhen 518060, China

^3^ Key Laboratory for Algal Biology, Institute of Hydrobiology, Chinese Academy of Sciences, Wuhan 430072, China

^4^College of Life Sciences, University of Chinese Academy of Sciences, Beijing, China.

^§^ Co-first author

* Corresponding author E-mail address: [Danxianghan@ihb.ac.cn](mailto:Danxianghan@ihb.ac.cn) (D. H.)


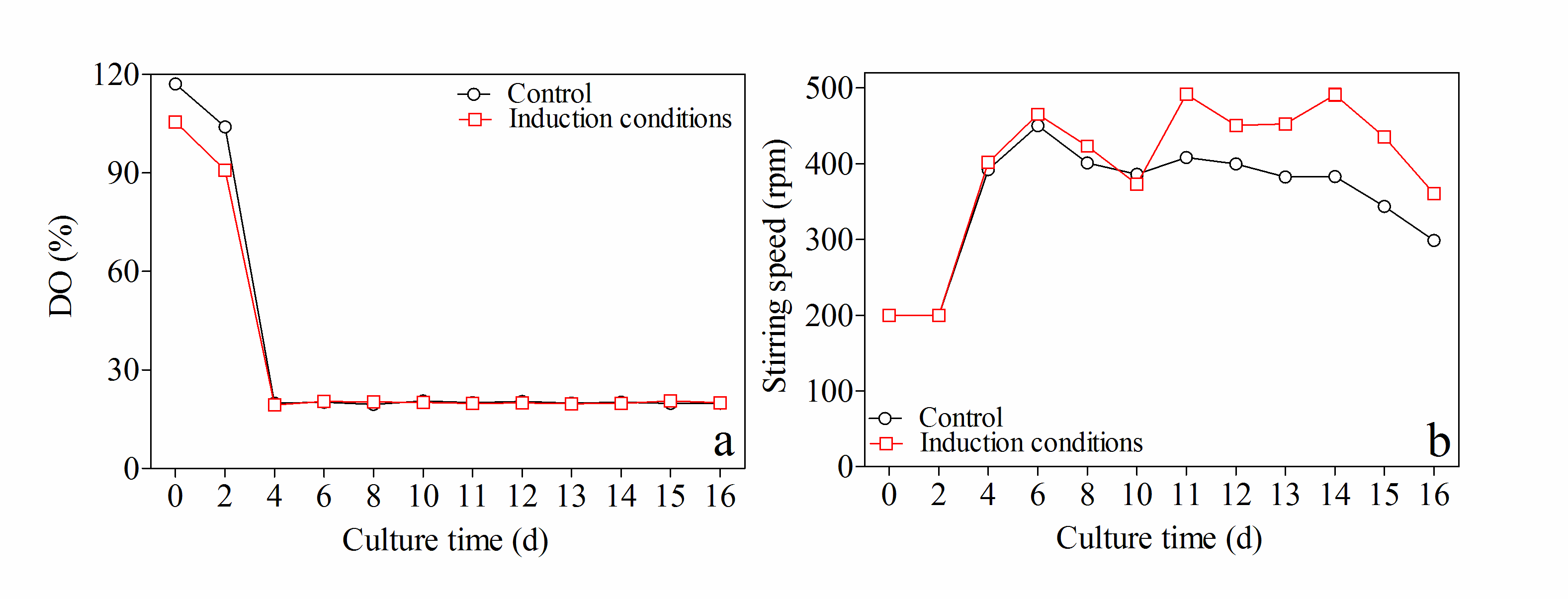
**Figure. 1S.** Heterotrophic culture of *C. zofingiensis* in 7.5 L fermentor with induction conditions. Time courses of dissolved oxygen (a) and stirring speed (b). Induction conditions, the C/N ratio of the feeding medium change to 180:1 with the addition of 10 mg·L^-1^ GA and 200 mM NaCl at the end of 10 days.

**Table S1**

Physico-chemical properties of phytohormones

| Type | Phytohormones | Abbreviation | Cosolvents | Molecular Formula | CAS Reg. No | Molecular mass |
| --- | --- | --- | --- | --- | --- | --- |
| Ethylene precursor | 1-aminocyclopropane-1-carboxylic acid | ACC | Water | C_4_H_7_NO_2_ | 22059-21-8 | 101.104 |
| Gibberellin | Gibberellic acid (GA_3_) | GA | Ethanol | C_19_H_22_O_6_ | 77-06-5 | 346.37 |
| Auxins and synthetic analogs | Indole-3-acetic acid | IAA | Ethanol | C_10_H_9_NO_2_ | 87-51-4 | 175.18 |
|  | Indole-3-propioponic acid | IPA | Ethanol | C_11_H_11_NO_2_ | 830-96-6 | 189.21 |
|  | Indole-3-butyric acid | IBA | Ethanol | C_12_H_13_NO_2_ | 133-32-4 | 203 |
|  | 1-Naphthylacetic acid | NAA | Ethanol | C_12_H_10_O_2_ | 86-87-3 | 186 |

**Table S2**

Factors and levels for the L_16_ orthogonal experiment

| Factors | Level 1 | Level 2 | Level 3 | Level 4 |
| --- | --- | --- | --- | --- |
| A:C/N | 140:1 | 180:1 | 220:1 | 280:1 |
| B:GA concentration (mg·L^-1^) | 10 | 50 | 100 | 150 |
| C:NaCl concentration (mM) | 100 | 200 | 400 | 600 |
